# Non-high-density lipoprotein cholesterol to high-density lipoprotein cholesterol ratio associated with psoriasis: a cross-sectional study

**DOI:** 10.3389/fmed.2024.1514275

**Published:** 2024-12-06

**Authors:** Xinyan Liu, Keou Li, Tingxuan Huang, Hongfeng Tang

**Affiliations:** Department of Dermatology, Shunde Hospital, The First People's Hospital of Shunde, Southern Medical University, Foshan, China

**Keywords:** NHHR, psoriasis, cardiovascular disease, NHANES, cross-sectional study

## Abstract

**Objective:**

Individuals with psoriasis face significant physiological and psychological burdens, and their compliance and satisfaction with medication regimens are alarmingly low. In recent years, the comorbidity of psoriasis has become a research focus. This study aims to explore the potential correlation between psoriasis and the non-high-density lipoprotein cholesterol to high-density lipoprotein cholesterol ratio (NHHR).

**Methods:**

Data from 17,941 participants of the National Health and Nutrition Examination Survey (NHANES) spanning two intervals, 2003–2006 and 2009–2014, served as the foundation for this analysis. We used weighted logistic regression, stratified analysis, and restricted cubic spline (RCS) curve fitting to validate potential associations between NHHR and psoriasis risk.

**Results:**

In this investigation, the analysis of three different models highlighted elevated levels of the NHHR as a consistent risk factor for psoriasis. After adjusting for potential confounders, a positive correlation was observed between NHHR and the prevalence of psoriasis (OR = 1.08, 95% CI: 1.01, 1.15, *p* < 0.05). Subgroup analysis and interaction tests were conducted to determine if variables such as age, gender, race/ethnicity, education level, smoking status, alcohol consumption, diabetes, coronary heart disease, and stroke impact the association between NHHR and psoriasis. The findings indicated no significant modification of the NHHR-psoriasis link by these factors, as all interaction *p*-values were above 0.05. The RCS analysis uncovered a nonlinear relationship between psoriasis and the NHHR (*p* = 0.0176).

**Conclusion:**

Statistical analysis confirms a significant correlation between the NHHR and the development of psoriasis, suggesting that NHHR may serve as a novel marker for predicting psoriasis risk. This correlation also provides insights for early health management strategies.

## 1 Introduction

Globally, psoriasis is recognized as a prevalent chronic inflammatory skin condition linked to immune system reactions, affecting about 2–4% of the population (1, 2). Notably, the latest Global Burden of Disease study indicates a rising incidence of psoriasis among individuals aged 15 to 39, imposing a substantial psychological burden on adolescents and young adult (3). Additionally, older patients face severe physical disability and a significantly increased risk of cardiovascular mortality due to comorbid conditions such as psoriatic arthritis and cardiovascular disease (4–6). Identifying new biomarkers for psoriasis risk assessment is crucial to enable early interventions and reduce the disease’s overall effects.

Research consistently shows that psoriasis patients often exhibit reduced High-Density Lipoprotein Cholesterol (HDL-C) and heightened concentrations of triglycerides and low-density lipoprotein cholesterol (LDL-C) (7, 8). However, the link between psoriasis and dyslipidemia continues to be a contentious topic. Recently, the understanding of psoriasis has expanded beyond its classification as just a skin condition. Psoriasis is now understood to be intimately linked with metabolic syndrome, cardiovascular disease, and inflammatory bowel disease, highlighting its status as a systemic condition accompanied by various comorbidities (9, 10). The non-high-density lipoprotein cholesterol to high-density lipoprotein cholesterol ratio (NHHR) provides a more thorough and precise evaluation compared to assessments based on single lipid level (11). The link between the NHHR and psoriasis remains inadequately examined. This study is designed to probe the potential correlation between these variables, illuminating the dynamics between lipid metabolism and the pathophysiology of psoriasis.

## 2 Methods

### 2.1 Data source and population

**Figure 1:**
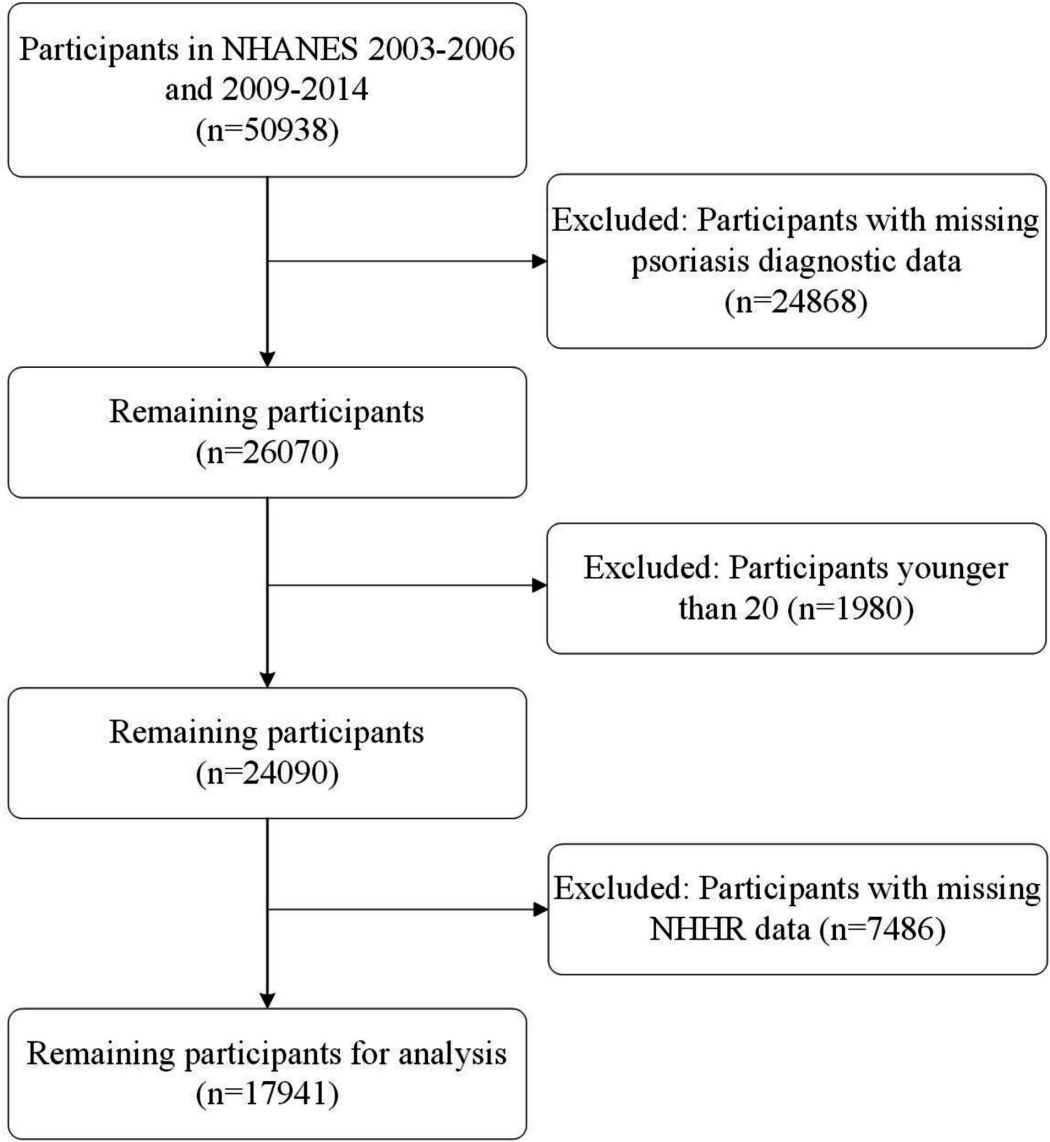
Flow chart of the study.

NHANES is a continuous and representative national survey program dedicated to assessing the health and nutritional status of the U.S. population. The program has obtained approval from the Research Ethics Review Board of the U.S. National Center for Health Statistics, and it ensures that all participants provide their informed consent prior to their involvement in the study. Our analysis is composed of cross-sectional samples from NHANES and encompassed data from five cycles (2003–2004, 2005–2006, 2009–2010, 2011–2012, and 2013–2014), wherein review information on psoriasis was accessible. Initially, we had a pool of 50,938 participants. Nevertheless, individuals younger than 20 years old and those lacking complete data on psoriasis or NHHR were systematically excluded from our analysis. After this rigorous screening process, a total of 17,941 participants were included in the cross-sectional analysis (Figure 1).

### 2.2 Evaluation of NHHR

This research focuses on the NHHR, a developing lipid index that provides new understanding into the risk of atherosclerosis. Non-High-Density Lipoprotein Cholesterol (Non-HDL-C) is ascertained by the simple subtraction of HDL-C levels from the overall total cholesterol (TC).

### 2.3 Psoriasis diagnosis

In this research, psoriasis serves as the primary outcome variable. The NHANES survey gathered essential data by querying participants with the question, “Have you ever been told by a doctor or healthcare provider that you have psoriasis?” Those who answered “Yes” were categorized as psoriasis patients, and all others were excluded.

### 2.4 Covariates

The research accounted for various factors potentially influencing psoriasis prevalence. Covariates in this study included age, gender, race/ethnicity, marital status, educational attainment, poverty income ratio (PIR), marital status, educational attainment, TC, HDL-C, smoking status, alcohol drinking, diabetes, coronary heart disease and stroke. Detailed information on these covariates is presented in Supplementary Table S1.

### 2.5 Statistical analysis

Our study methodology incorporated the use of sampling weights as prescribed by NHANES guidelines to ensure that the data were correctly balanced and representative. We presented continuous variables by stating the mean and standard error (SE), while categorical variables were shown using frequencies and percentages. Our study initially categorized NHHR levels into tertiles (Q1, Q2, and Q3) as categorical variables, with Q1 as the baseline group for analysis. For continuous variables, we analyzed by weighted linear regression models. For categorical variables, we analyzed differences between groups by weighted chi-square tests. The relationship between NHHR and psoriasis was analyzed using multivariate logistic regression models. We used the NHHR as an independent variable in both continuous and tertile forms. This study investigated the independent relationship between NHHR and psoriasis using three different models. Model 1 was basic, with no adjustments. In Model 2, adjustments were made for demographic factors such as age, sex, and race/ethnicity. In Model 3, and most detailed, model further included controls for a variety of socio-economic and health-related variables, specifically educational level, marital status, the poverty income ratio, along with personal behaviors and medical history encompassing smoking, alcohol consumption, diabetes, coronary heart disease, and stroke. In addition, we used restricted cubic spline (RCS) regression analysis to assess whether there was a nonlinear relationship between NHHR levels and the prevalence of psoriasis. Finally, we used stratified multivariate regression models for subgroup analyses. Stratified factors included age, gender, race/ethnicity, marital status, education level, smoking (smokers vs. non-smokers), alcohol consumption (drinkers vs. non-drinkers), and presence of diabetes mellitus, coronary heart disease, or stroke (all categorized as present or absent). This comprehensive approach allows for a careful examination of factors that may modulate the association between psoriasis and NHHR. Statistical analysis was conducted using version 4.1.3 of the R software. A *p*-value of less than 0.05 was considered statistically significant.

## 3 Results

### 3.1 Characteristics of the study participants

In this research, the participant group includes 17,941 adults, averaging 44.61 years old with a standard deviation of 15.62 years. The baseline characteristics of this study population, categorized into three NHHR tertiles, are detailed in Table 1. Quartiles are defined as follows: Q1 (<2.175), Q2 (2.175–3.280), Q3 (>3.280). Notable differences are observed across various demographics including age, gender, race/ethnicity, education level, marital status, PIR, smoking, diabetes. Intriguingly, participants with higher NHHR ratios tend to be males, older, and exhibit lower levels of HDL-C alongside higher levels of TC.

**Table 1:** Basic characteristics of U.S. adult participants by psoriasis.

Characteristic	Quartiles of NHHR			*p*-value
	Q1 (<2.175) *N* = 5,965	Q2 (2.175–3.280) *N* = 5,996	Q3 (>3.280) *N* = 5,980	
Age (years), mean ± SD	44.04 ± (17.02)	44.85 ± (15.86)	44.92 ± (13.87)	0.002
Sex, *n* (%)				<0.001
Male	2,163 (35%)	2,783 (47%)	3,826 (66%)	
Female	3,802 (65%)	3,212 (53%)	2,155 (34%)	
Race/ethnicity, *n* (%)				<0.001
Mexican American	718 (6.4%)	912 (8.1%)	1,119 (9.9%)	
Other Hispanic	366 (3.8%)	461 (4.8%)	536 (5.6%)	
Non-Hispanic White	2,767 (70%)	2,792 (70%)	2,888 (70%)	
Non-Hispanic Black	1,531 (13%)	1,263 (11%)	946 (8.1%)	
Other Race	583 (6.7%)	567 (6.2%)	492 (6.0%)	
Education level, *n* (%)				<0.001
Less than high school	1,125 (13%)	1,349 (15%)	1,542 (17%)	
High school	1,216 (19%)	1,343 (22%)	1,495 (25%)	
More than high school	3,624 (68%)	3,303 (63%)	2,944 (57%)	
Marital status, *n* (%)				<0.001
Married/Living with partner	3,279 (60%)	3,636 (65%)	3,945 (68%)	
Unmarried/Separated/widowed	2,686 (40%)	2,359 (35%)	2036 (32%)	
PIR, *n* (%)				<0.001
<1.3	1,697 (19%)	1859 (21%)	2080 (23%)	
1.3–3.5	2,162 (35%)	2,181 (35%)	2,134 (36%)	
>3.5	2,106 (46%)	1955 (44%)	1767 (41%)	
TC (mg/dL), mean ± SD	175.44 ± (34.27)	191.81 ± (35.60)	219.38 ± (42.89)	<0.001
HDL-C (mg/dL), mean ± SD	67.40 ± (15.74)	52.23 ± (10.22)	40.78 ± (8.22)	<0.001
Smoking status, *n* (%)				<0.001
Yes	2,480 (43%)	2,596 (44%)	2,992 (50%)	
No	3,485 (57%)	3,399 (56%)	2,989 (50%)	
Alcohol drinking, *n* (%)				0.077
Yes	4,408 (79%)	4,271 (77%)	4,437 (78%)	
No	1,557 (21%)	1724 (23%)	1,544 (22%)	
Diabetes, *n* (%)				<0.001
Yes	552 (6.8%)	665 (7.8%)	681 (9.4%)	
No	5,413 (93%)	5,330 (92%)	5,300 (91%)	
Coronary heart disease, *n* (%)				0.424
Yes	201 (2.8%)	190 (2.6%)	166 (2.4%)	
No	5,764 (97%)	5,805 (97%)	5,815 (98%)	
Stroke, *n* (%)				0.378
Yes	184 (2.4%)	160 (2.0%)	164 (2.1%)	
No	5,781 (98%)	5,835 (98%)	5,817 (98%)	

Mean (SD) for continuous variables: the *p* value was calculated by the weighted Students T-test. Percentages (95% CI) for categorical variables: the *p* value was calculated by the weighted chi-square test. PIR, family income-to-poverty ratio. TC, total cholesterol. HDL-C, high-density lipoprotein cholesterol. NHHR, non-high-density lipoprotein cholesterol to high-density lipoprotein cholesterol ratio.

### 3.2 NHHR level and psoriasis

**Figure 2:**
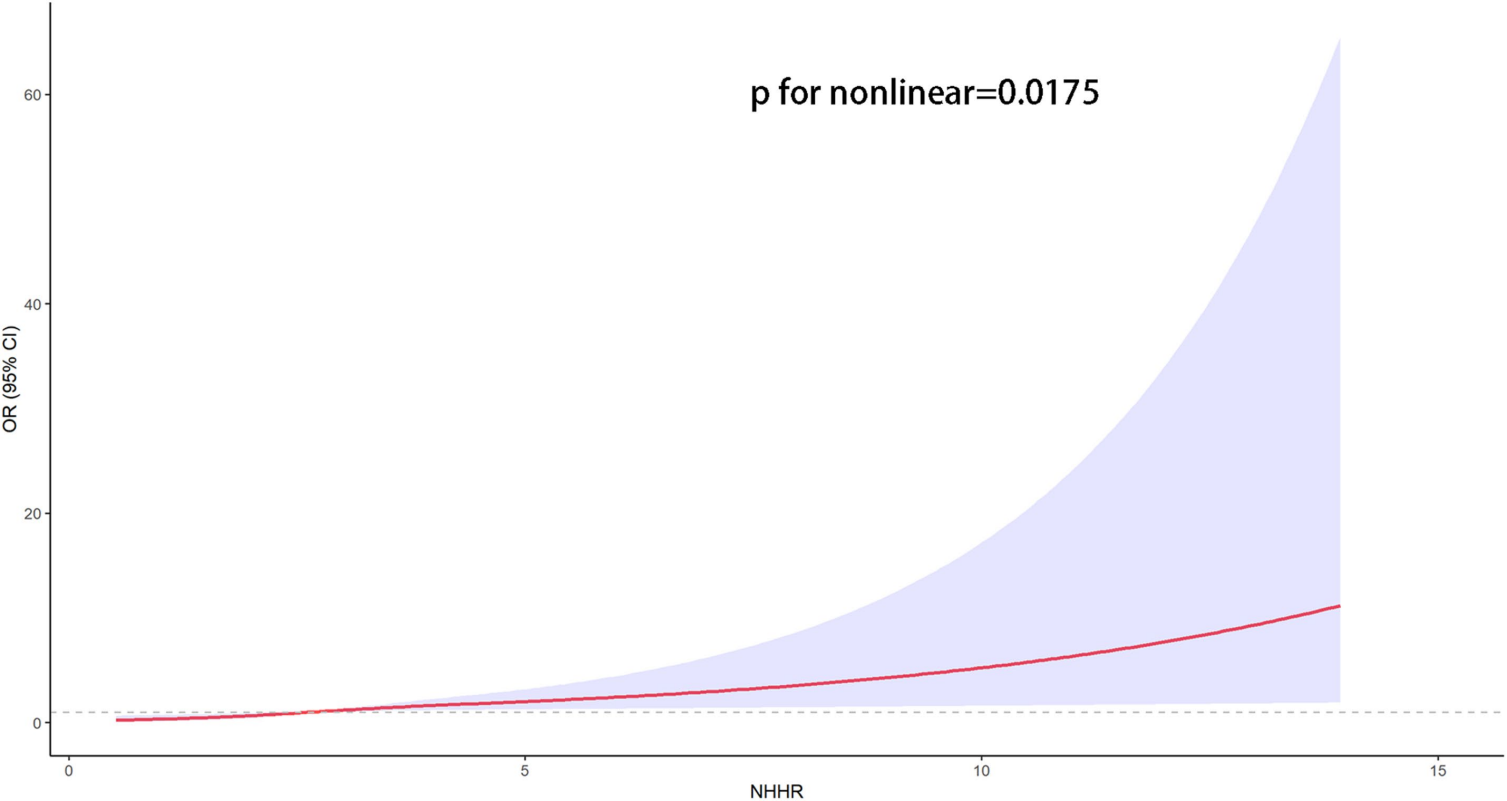
The restricted cubic spline curves for the association between the NHHR and psoriasis. Models adjusted for age, gender, race/ethnicity, marital status, educational attainment, PIR, marital status, educational attainment, TC, HDL-C, smoking status, alcohol drinking, diabetes, coronary heart disease and stroke. The solid red line indicates a smooth curve fit between the variables. The purple portion indicates the fitted 95% confidence interval. 95% CI, 95% confidence interval; OR, odds ratio; *p* < 0.05 was considered statistically significant.

The association between NHHR and psoriasis is illustrated in Table 2. To sum up, higher NHHR levels are correlated with a greater likelihood of psoriasis in all analyzed models (*p* for trend <0.05). After controlling for multiple variables, the odds ratios (OR) and their respective 95% confidence intervals (CIs) for individuals Q2 and Q3 quartiles of NHHR in comparison to the lowest quartile were 1.42 (95% CI: 1.06 to 1.92) and 1.47 (95% CI: 1.13 to 1.92), respectively. To further explore the intricate relationship between NHHR and the risk of psoriasis, we employed a restricted cubic spline curve analysis, with the results illustrated in Figure 2. We found a linear relationship between NHHR and the risk of psoriasis (*p* for Nonlinear = 0.0175).

**Table 2:** Association between NHHR and psoriasis risk.

Variables	Model 1		Model 2		Model 3	
	OR (95%CI)	*p* value	OR (95%CI)	*p* value	OR (95%CI)	*p* value
NHHR	1.07 (1.01,1.15)	0.030	1.08 (1.01,1.15)	0.024	1.08 (1.01,1.15)	0.030
Q1	Ref		Ref		Ref	
Q2	1.39 (1.03,1.87)	0.031	1.40 (1.04,1.89)	0.029	1.42 (1.06,1.92)	0.022
Q3	1.42 (1.10,1.84)	0.008	1.45 (1.12,1.88)	0.005	1.47 (1.13,1.92)	0.005
*p* for trend	0.007		0.005		0.005	

Model 1 incorporated no adjustments. Model 2 included controls for sex, age, and race/ethnicity. Model 3 was comprehensive, adjusting for sex, age, race/ethnicity, poverty income ratio, educational level, marital status, TC, HDL-C, alcohol consumption, smoking habits, diabetes, coronary heart disease, and stroke. *p* < 0.05 was considered statistically significant.

### 3.3 Subgroup analyses

**Figure 3:**
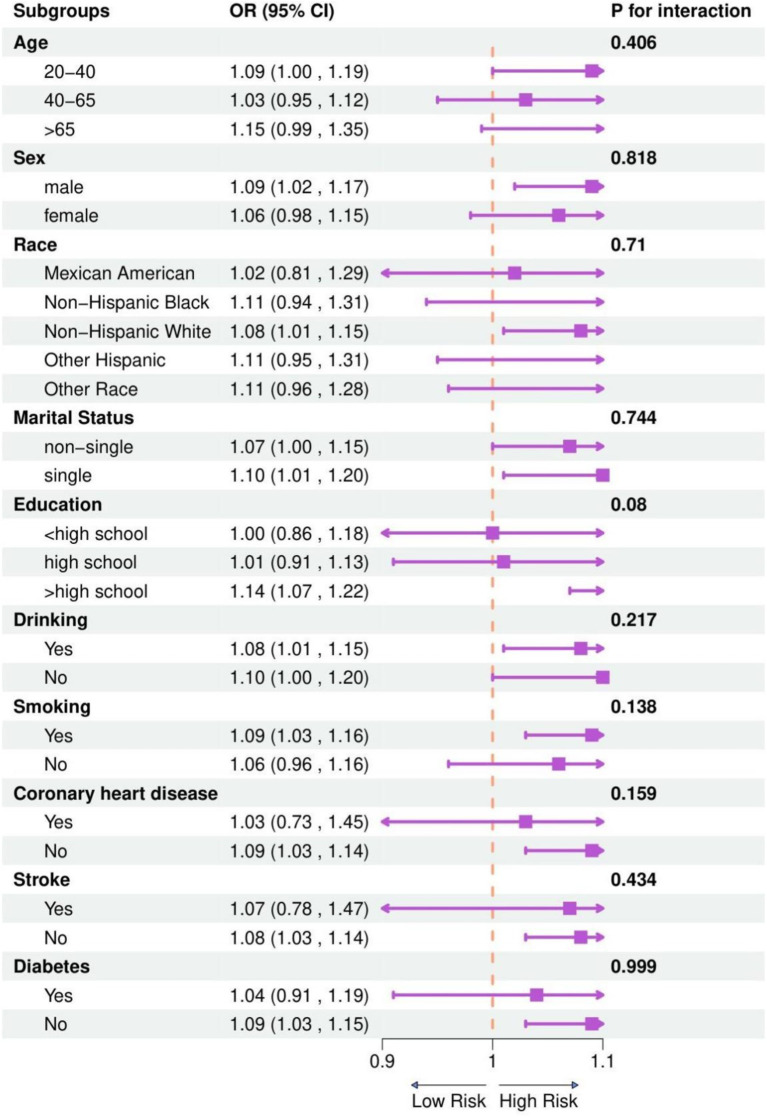
Subgroup analysis between NHHR and psoriasis. Analyses were adjusted for age, gender, race/ethnicity, marital status, educational attainment, PIR, marital status, educational attainment, TC, HDL-C, smoking status, alcohol drinking, diabetes, coronary heart disease and stroke. 95% CI, 95% confidence interval; OR, odds ratio; *p* < 0.05 was considered statistically significant.

In exploring the link between NHHR and psoriasis risk variability among diverse groups, we performed segmented analyses. This analysis integrated a variety of demographic and lifestyle elements such as age, gender, race/ethnicity, education level, smoking and drinking behaviors, and the presence of diabetes, coronary heart disease and stroke. Figure 3 clearly depicts that the outcomes remained uniform across various subgroups, showing no significant interactive effects.

## 4 Discussion

This study utilizes large-scale population-based data from NHANES to elucidate the relationship between NHHR and psoriasis. Analysis through three distinct logistic regression models uniformly reveals a positive association between NHHR and increased psoriasis risk. The RCS curve elucidated a nonlinear dynamic between NHHR and psoriasis risk, supporting our findings. Individuals exhibiting elevated NHHR levels are at an increased risk for psoriasis. Due to its accessibility, NHHR holds promise as a novel biomarker for assessing the risk of psoriasis, aiding in the development of effective public health strategies to reduce the incidence and burden of the disease.

Once solely classified as a skin disorder, recent research has expanded the understanding of psoriasis, identifying its connection with a variety of other health conditions. These include metabolic syndrome, cardiovascular disease, psoriatic arthritis, autoimmune disorders, and psychiatric illnesses (12–14). Studies have shown that psoriasis is an independent risk factor for cardiovascular disease (CVD) (15). In addition, a meta-analysis of 14 cohorts revealed that among patients with severe psoriasis, the risk ratio for CVD mortality was 1.37 (95% CI: 1.17–1.60), myocardial infarction (MI) was 3.04 (95% CI: 0.65–14.35), and stroke was 1.59 (95% CI: 1.34–1.89) (16). The results underscore the complexity of managing psoriasis, highlighting the need for both preventative measures and a comprehensive treatment approach to effectively handle the condition.

NHHR has emerged as a new, precise measure for evaluating the risk of cardiovascular conditions (17, 18). Recent research supports its credibility in predicting the onset of diabetes (19), osteoporosis (20), and suicidal ideations (21). In the case of psoriasis, it has been identified that specific cytokines (TNF-*α*, IL-17, and IL-22) secreted by subsets of T-cells are responsible (22). These cytokines trigger the excessive growth of keratinocytes and provoke inflammatory reactions (23). Research consistently indicates a tendency toward higher LDL and TG levels and lower HDL levels among psoriasis patients (24, 25). Yet, a definitive link between psoriasis and lipid levels in the blood remains elusive in some studies (26). The exact processes that intertwine psoriasis with lipid abnormalities are not fully understood, but genetics and shared inflammatory mechanisms are likely influential. HDL plays a pivotal role in moderating inflammation and oxidative stress, affecting the function of various immune cells such as dendritic cells, monocytes, macrophages, T cells, and B cells (25, 27). HDL impedes lipid peroxidation through its components, including apolipoprotein A-I (apoA-I), Paraoxonase-1 (PON-1), and lecithin-cholesterol acyltransferase (LCAT), demonstrating its antioxidant properties (7, 28). In mouse models used to study psoriasis, which is induced by imiquimod, elevated levels of phospholipid transfer protein (PLTP) and cholesterol ester transfer protein (CETP) exacerbate the condition and increase the risk of atherosclerosis (29). Mehta et al. found that HDL efflux capacity is reduced in patients with psoriasis (30). Michael et al. discovered modifications in both the composition and functionality of HDL among psoriasis patients, characterized by diminished levels of apoA-I and reduced activity of the PON-1 enzyme (28). Furthermore, a decrease in PON-1 enzyme activity was identified in these patients. Additionally, Batuca’s research highlights that in psoriasis patients, the existence of antibodies targeting HDL and apoA-I leads to impaired HDL function (31). These antibodies are also implicated in the development of atherosclerosis. At the same time, the application of biologic agents for the treatment of psoriasis can restore the composition and function of HDL (32). These studies indirectly confirm our results, suggesting a potential link between lipid metabolism and psoriasis.

## 5 Strengths and limitations

This study is the first to explore the possible relationship between NHHR and psoriasis, providing a new scientific basis for the complex association between lipid metabolism and autoimmune skin diseases, as well as opening up new strategic pathways for future precision prevention and treatment of psoriasis. However, the present study acknowledges a few inherent limitations. To begin with, our reliance on self-reported questionnaires for diagnosing psoriasis introduces a potential recall bias. Additionally, the absence of longitudinal NHHR data correlated with the fluctuation of psoriasis symptoms restricts our ability to use NHHR as a marker for monitoring disease progression and therapeutic efficacy over time. Lastly, causality between NHHR levels and the onset of psoriasis remains undetermined, necessitating future prospective research to substantiate this association.

## 6 Conclusion

The findings suggest that a higher level of NHHR is associated with an increased risk of psoriasis in adults in the U.S. The NHHR may serve as a potential biomarker for assessing psoriasis risk and provide new insights into the role of lipid metabolism in psoriasis. It is expected to contribute to the prevention of psoriasis through lipid management in the future.

## Data Availability

Publicly available datasets were analyzed in this study. This data can be found at: https://www.cdc.gov/nchs/nhanes/index.htm.
