# Supplementary material for: Non-high-density lipoprotein cholesterol to high-density lipoprotein cholesterol ratio associated with psoriasis: a cross-sectional study
Source: Front Med (Lausanne). 2024 Dec 6;11:1514275. doi: 10.3389/fmed.2024.1514275 (PMC11659000; doi:10.3389/fmed.2024.1514275)
Supplement: Supplementary file 1 [file Table_1.DOCX]

**Table S1.** Definition of variables involved in this study.

| Variables | Description in NHANES |
| --- | --- |
| Age | Age was obtained from a self-reported questionnaire |
| Gender | Male and Female |
| race/ethnicity | Use of racial categorization in the NHANES database：Mexican American, Other Hispanic, Non-Hispanic Black, Non-Hispanic White, Other Race |
| Educational level | Below high school, High School, or above |
| Marital status | Married/ Living with partner,Unmarried/ Separated/ widowed |
| PIR | less than 1.3, between 1.3 and 3.5, and more than 3.5 |
| TC (mg/dL), HDL-C(mg/dL) | TC and HDL-C were collected at the mobile exam center. |
| Smoking status | Self-adjustment: Had smoked at least 100 cigarettes in entire life? |
| Alcohol drinking | Self-adjustment: Had at least 12 alcoholic drinks in a year? |
| Diabetes | Diabetes was defined as a history of previous diabetes, HbA1c level ≥6.5%, or fasting blood glucose level ≥126 mg/dL |
| Coronary heart disease | Yes indicates having been told by a doctor or health professional that you have coronary heart disease, denial is answered No. |
| Stroke | Yes indicates having been told by a doctor or health professional that you have stroke, denial is answered No. |

PIR, Ratio of family income to poverty.TC, total cholesterol. HDL-C, high-density lipoprotein cholesterol.
